# Correlates of Acceptance of Wealth Inequality: A Moderated Mediation Model

**DOI:** 10.3389/fpsyg.2019.00900

**Published:** 2019-04-25

**Authors:** Grand H.-L. Cheng, Darius K.-S. Chan, Dannii Y. Yeung

**Affiliations:** ^1^Centre for Ageing Research and Education, Duke-NUS Medical School, National University of Singapore, Singapore, Singapore; ^2^Department of Psychology, The Chinese University of Hong Kong, Shatin, Hong Kong; ^3^Department of Social and Behavioural Sciences, City University of Hong Kong, Kowloon Tong, Hong Kong

**Keywords:** wealth inequality, income, upward mobility, future time perspective, collective action

## Abstract

Wealth inequality is a prevalent social issue. The present study focuses on acceptance of wealth inequality, and considers personal income, perceived upward mobility, and future time perspective as its antecedents, and collective action intention as its outcome. With reference to the social identity literature and socioemotional selectivity theory, we posit a conditional indirect effect of income on collective action intention through acceptance of wealth inequality: only when mobility and future time perspective are relatively high, higher income is associated with higher acceptance of wealth inequality and in turn, lower collective action intention. Moderated mediation findings, based on territory-wide phone survey data from 866 Hong Kong adults, supported this prediction. This work indicates the relevance of an individual-level instrumental perspective to the understanding of acceptance of wealth inequality as well as collective action intention.

## Introduction

Societal-level analyses have documented that wealth inequality^1^ brings about a range of undesirable social outcomes such as lower happiness, worse health, and increased mistrust (Pickett and Wilkinson, 2015; Buttrick and Oishi, 2017). Protests against wealth inequality have sprouted around the world, and a notable example is Occupy Wall Street in New York in 2011. Given that wealth inequality fuels social discontent and instability, it certainly warrants research attention.

Social scientists have paid increasing attention to acceptance of wealth inequality (AWI), or evaluation of the existing wealth inequality in the society (e.g., whether thinking that wealth inequality is acceptable) (Savani and Rattan, 2012; Shariff et al., 2016). A line of research has considered income as a determinant of AWI. For instance, Hadler (2005) posited a positive relation between income and AWI (the structural position thesis; see also Gijsberts, 2002). At the same time, some research has suggested that AWI may influence collective action against wealth inequality (Hennes et al., 2012; Jost et al., 2012).

Examining both antecedents and consequences of a psychological construct is vital to theory development (Meyer et al., 2002). In this study of AWI, we consider personal income and collective action intention (CAI) as an antecedent and a consequence of AWI, respectively. That is, we establish a framework that describes AWI as a mediator of the income–CAI relation. This attempt is relevant to the longstanding discussion of the impact of income on collective action (Wakslak et al., 2007; Kugler et al., 2010).

In addition, we address the boundary condition for the income–AWI–CAI association, or how the indirect effect of income on CAI via AWI varies. Based on the social identity literature (Tajfel and Turner, 1979; Hogg, 2005) and socioemotional selectivity theory (Carstensen, 1991, 2006; Carstensen et al., 1999), we reason that perceived upward mobility and future time perspective (FTP) may moderate the relationship between income and AWI. In studying these two moderating effects, we provide a detailed picture of the income–AWI–CAI association.

### Income–AWI–CAI Association

One perspective that is relevant to AWI is the system justification theory (Jost and Hunyady, 2005). This theory argues that a human motivation is to rationalize the way things are. People vary in the endorsement of system justification ideologies, which are considered as individual differences or personality dimensions (Pratto et al., 1994; Kugler et al., 2010). An example of system justification ideologies is social dominance orientation, which is a “general attitudinal orientation toward intergroup relations, reflecting whether one generally prefers such relations to be equal, versus hierarchical,” and the “extent to which one desires that one’s ingroup dominate and be superior to out-groups” (Pratto et al., 1994, p. 742). Another example is economic system justification (Jost and Thompson, 2000). A high (low) degree of this orientation means that one thinks (does not think) that economic inequality is natural and legitimate. As suggested by the system justification theory, some people just tend to think that social arrangements including the economic system are legitimate, and therefore have high levels of AWI (Starmans et al., 2017).

Another body of research focuses on the role of personal income in AWI (Gijsberts, 2002; Hadler, 2005). Wealth inequality is by definition concerned with income. All else being equal, higher personal income implies a higher social status, particularly for adults (Ellis et al., 2018). Wealth inequality literally means an existence of profound status difference in the society. Personal income may predict AWI, and the social identity literature (Tajfel and Turner, 1979; Hogg, 2005) provides some insights into this association.

The social identity literature includes a set of interconnected concepts about intragroup processes and intergroup relations, including subgroup relations within a superordinate group (Hogg, 2005). One of its central premises is that membership in a high status group can promote self-esteem and satisfy self-enhancement motives. Hence, one strives for positive intergroup distinctiveness through strategic social comparison (Tajfel and Turner, 1979). This assertion suggests that while people in high status may endorse status difference, people in low status should tend to reject it. Past studies have shown that higher income and status entail higher endorsement of inequality and less support for redistribution (Wakslak et al., 2007; Kugler et al., 2010; Brown-Iannuzzi et al., 2017; Jetten et al., 2017). Extending these insights to the present investigation, we posit that personal income should be positively associated with AWI; rich people should have higher levels of acceptance of existing wealth inequality than poor people.

Next, we discuss the implication of AWI. While many outcome variables could be considered, here, we examine CAI. Collective action (e.g., demonstration, signing a petition) refers to an act that targets to foster or hinder social change, for a social good or against a social ill (van Zomeren and Iyer, 2009; van Zomeren, 2013). For wealth inequality which is an undesirable social phenomenon, it is useful to understand what may modulate the collective action against it.

Some collective action studies address how group-related factors such as group efficacy affect CAI (van Zomeren et al., 2011; van Zomeren, 2013). Another stream examines how individual disgruntlements are transformed into collective action, and this transformation reflects an attitude-behavior relation (Klandermans, 1997; Hogg, 2005; van Zomeren et al., 2008): when individuals hold a negative evaluation of a social issue, they may engage in collective action to tackle this issue. For instance, appraised illegitimacy of discrimination can motivate the corresponding collective action (Iyer and Ryan, 2009). Specifically, on wealth inequality, Americans who perceived the local economic system as unfair were found to be supportive of the collective action of Occupy Wall Street, and willing to take part in disruptive protest against the United States government’s Wall Street bailout (Hennes et al., 2012; Jost et al., 2012). This evidence suggests that higher AWI entails lower CAI.

Collectively, we hypothesize that AWI mediates the relation between income and CAI; higher income is associated with higher AWI and in turn, lower CAI (Hypothesis 1; H1). This speculation is consistent with the previous findings that higher income leads to weaker support for economic redistribution through lower opposition to inequality (Wakslak et al., 2007; Kugler et al., 2010).

### Moderating Effects and Conditional Indirect Effect

So far, we have reasoned personal income as a factor affecting AWI. We further posit that perceived upward mobility and FTP may play a role in AWI. Supporting evidence, if found, will consolidate our instrumental perspective on AWI.

In addition to income, perceived upward mobility is another construct related to social status (Jetten et al., 2008). According to the social identity literature (Tajfel and Turner, 1979; Hogg, 2005), individuals strive to belong to a high-status group which provides them with a positive social identity. When perceiving low levels of upward mobility, individuals may engage in social competitions against the high-status groups (Mummendey et al., 1999). Indeed, wealth inequality research has reported that individuals who perceive that they cannot move up the social hierarchy are less tolerant of inequality (Shariff et al., 2016). Applying these insights to the relation between income and AWI, it is plausible that rich people do not accept wealth inequality if they perceive that they cannot further their social status. Alternatively stated, rich people may accept wealth inequality only when they perceive that they can still enhance their social status. Overall, the positive association of income with AWI may be moderated by mobility: it should be more profound among individuals with high mobility than those with low mobility (two-way interaction between income and mobility) (H2).

People who are better off in terms of personal income and perceived upward mobility should show high levels of AWI. Nevertheless, people vary in instrumental needs. Those who have weaker instrumental needs should be less sensitive to and less affected by their instrumental status. It follows that the effects of income and mobility on AWI may be rather weak among these people.

We operationalize instrumental needs in terms of FTP, or perceived time left in life. FTP is a central construct of the socioemotional selectivity theory (Carstensen, 1991, 2006; Carstensen et al., 1999), a lifespan psychology perspective. Lifespan scholars argue that individuals manage their social and psychological resources to achieve successful development. For instance, Baltes’ model of selective optimization with compensation (Baltes and Baltes, 1990) asserts that people engage in behaviors that allow continued growth, and compensate for lost functioning through adaptation. Older age is characterized by limiting chances and varied losses and hence, aging individuals become selective, such as focusing on certain social networks. Similarly, the socioemotional selectivity theory proposes that with age, individuals shift their priority from meeting instrumental goals (e.g., exploring the physical world) to meeting emotionally meaningful goals (e.g., maintaining close relationships).

Related to age, FTP is indicative of instrumental needs (Carstensen, 1991, 2006; Carstensen et al., 1999): when individuals perceive their amount of future time as increasingly limited, they focus more on emotionally meaningful goals, and less on instrumental goals such as individual competition and achievement. This assertion has received abundant empirical support (Henry et al., 2017). For instance, individuals with high FTP exhibited more career-related networking behaviors (Treadway et al., 2010). On the other hand, the effect of social work-related values on job outcomes were stronger among employees with low FTP (Yeung et al., 2013).

In this light, we propose the third hypothesis (H3) that FTP should qualify the aforementioned interaction effect between personal income and perceived upward mobility on AWI. Specifically, this interaction effect may only emerge among people with open-ended FTP as these individuals have stronger instrumental values, and are concerned more about self-interest and achievement. In contrast, people with limited FTP have weaker instrumental needs. Among them, income may be unrelated to AWI, regardless of levels of mobility. That is, it is possible that income is positively related to AWI only when the levels of mobility and FTP are relatively high.

Previously, we have posited that AWI, as a consequence of personal income, should channel the effect of income on CAI. Now, with H3 that concerns a three-way interaction in place, a conditional indirect effect (Hayes, 2013) of income on CAI via AWI can be derived: only when the levels of mobility and FTP are relatively high, higher income is related to higher AWI and in turn, lower CAI (H4).

### The Present Study

**FIGURE 1:**
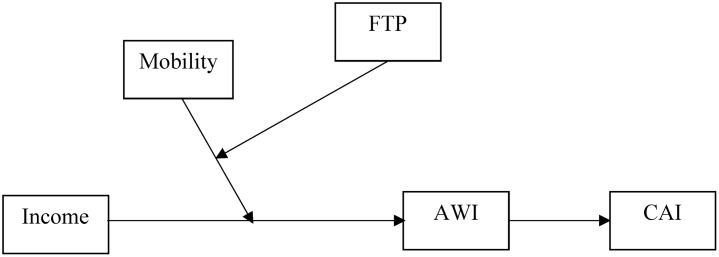
Conceptual framework. Income, personal income; Mobility, perceived upward mobility; FTP, future time perspective; AWI, acceptance of wealth inequality; CAI, collective action intention.

The current research examines a moderated mediation model (Hayes, 2013) of AWI that conceptualizes personal income as the main predictor, perceived upward mobility and FTP as the moderators, AWI as the mediator, and CAI as the final outcome (Figure 1). Our research context was Hong Kong. Gini coefficient is a measure of wealth inequality for a certain population, and it is often noted that a value greater than 0.4 suggests a risk of social unrest (The World Economic Forum, 2014). The figure was reported as 0.479 in the United States (United States Census Bureau, 2016), and 0.492 in the United Kingdom (United Kingdom Office of National Statistics, 2017). In Hong Kong, it was even higher, at 0.539 (Hong Kong Census and Statistics Department, 2017b). Hong Kong represents a relevant research context.

## Materials and Methods

### Participants

Data come from a Hong Kong government-funded project on local adult citizens’ social attitudes. After obtaining ethics approval of human subject research from the Chinese University of Hong Kong, we hired a local research laboratory to conduct a territory-wide phone survey (Cantonese was the medium of communication) for data collection. Stratified random sampling, with age as the selection variable, was adopted. The age range was 18–70 years. A total of 1,402 adults (42% response rate) participated in our survey. A focal variable of the present investigation was current personal income. The analytical sample (*N* = 866) involved employees (93.9%) and unemployed people (6.1%). Students, housewives, and retirees were excluded here. We did not have data on personal income of the latter groups; they were not asked to report their personal income as it is somewhat irrelevant.

### Measures

A higher score indicated a higher level of a construct. Items were prepared through back translation (Brislin, 1986).

#### Personal Income (Income)

Participants reported their current personal monthly income on an ordinal scale: 0 = HK$0 (unemployed; 6.1% of participants), 1 = below $5000 (4.1%), 2 = at least $5000 and below $10000 (12.4%), 3 = at least $10000 and below $20000 (30.7%), 4 = at least $20000 and below $30000 (21.0%), 5 = at least $30000 and below $40000 (9.9%), and 6 = $40000 or above (15.8%)^2^.

#### Perceived Upward Mobility (Mobility)

Mobility was measured by two items (1 = strongly disagree, 5 = strongly agree): “I have the opportunities to climb up the social ladder and improve my socioeconomic status,” and “Based on my abilities and experiences, I think I can improve my economic condition.” These items were adapted from Jetten et al.’s (2008) measure which assesses mobility of university students.

#### Future Time Perspective (FTP)

Carstensen and Lang (1996) developed a 10-item measure of FTP which has been widely utilized. To reduce the burden of participants and enhance the completion rate of the telephone survey, we chose three items of this scale based on a pilot study (a convenient sample of 200 adults): “I expect that I will set many new goals in the future,” “My future is filled with possibilities,” and “Most of my life lies ahead of me” (1 = strongly disagree, 5 = strongly agree). These three items had the highest item-total correlation and factor loading. It is also noteworthy that they are included in other shortened FTP measures (Zacher and Frese, 2009; Li, 2013).

#### Acceptance of Wealth Inequality (AWI)

Similar to Savani and Rattan (2012), we asked participants to report the extent to which they accepted factual statistics illustrating the existing wealth inequality in Hong Kong (1 = totally not accept, 5 = totally accept). The two factual statistics considered are: “According to the 2014 Census report on distribution of household income in Hong Kong, the top 8% of monthly household income is HKD 80,000 or above, while the bottom 11% of monthly household income is less than HKD 6,000,” and “According to the 2014 Census report on distribution of employee salaries in Hong Kong, the top 10% of monthly salary is HKD 37,000, while the bottom 10% of monthly salary is HKD 8000.”

#### Collective Action Intention (CAI)

Collective action intention was measured by two items (1 = strongly disagree, 5 = strongly agree): “I would participate in some form of collective action to stop wealth inequality in Hong Kong,” and “I would participate in raising our collective voice to stop wealth inequality.” These items were adapted from van Zomeren et al. (2004).

#### Covariates

Covariates included age (ordinal: 1 = 18–29 years, 2 = 30–39, 3 = 40–49, 4 = 50–59, 5 = 60–70), gender (dummy coded: 1 = male), marital status (dummy: 1 = married), education level (ordinal: 1 = below secondary, 2 = secondary, 3 = tertiary), identification with Hong Kong (two items, 1 = strongly disagree, 5 = strongly agree; adapted from Jetten et al., 2002), and past collective action against wealth inequality (dummy: 1 = yes).

### Analysis

We tested our hypotheses using Hayes’s (2013) SPSS MACRO (Model 11). Covariates were controlled for the prediction of AWI and CAI. We retained the continuous/ordinal nature of the predictors (income, mobility, FTP) and used their centered scores to examine moderating effects. A significant moderating effect means that the predictor–outcome association varies across levels of the moderator. The standard way to follow up significant moderating effects is simple slope analysis (Aiken and West, 1991). Here, we *illustrated* the three-way interaction (if found) with four “simple slopes”: for the income–AWI association when mobility and FTP were both “high” (1 *SD* above the means), when mobility was “high” and FTP was “low” (1 *SD* below the mean FTP), when mobility was “low” and FTP was “high,” and when mobility and FTP were both “low,” respectively. Note that for plotting reasons, we also referred to “high” and “low” income in the figure. We also tested the statistical significance of these four slopes or lines. Indirect effect is the product term of the coefficient of the predictor-mediator (in our case, income-AWI) association and that of the mediator-outcome (AWI-CAI) association. We applied bootstrapping (5000 samples) to evaluate the significance level of the (conditional) indirect effect because the assumption of a normal distribution of indirect effects is often violated (Geiser, 2013). A 95% bias-corrected bootstrap confidence intervals (CI) that excludes zero indicates a significant indirect effect. A significant conditional indirect effect means that the indirect effect varies across levels of the moderator. We *illustrated* it by reporting indirect effects at different combinations of levels of mobility and FTP. All these procedures are well-acknowledged and widely adopted (Aiken and West, 1991; Geiser, 2013; Hayes, 2013).

## Results

Table 1 reports the descriptive statistics and intercorrelation of the variables. We observed that, central to the present arguments, AWI was positively related to income (*r* = 0.16, *p* < 0.001) and mobility (*r* = 0.45, *p* < 0.001) at zero-order level. CAI, which was negatively correlated with AWI (*r* = -0.28, *p* < 0.001), had a negative relationship with income (*r* = -0.08, *p* = 0.025) and mobility (*r* = -0.22, *p* < 0.001).

**Table 1:** Descriptive statistics and correlations.

Variable		1	2	3	4	5	6	7	8	9	10	11
1.	Personal income	–										
2.	Perceived upward mobility	**0.27**	(0.76)									
3.	Future time perspective	**0.14**	**0.36**	(0.75)								
4.	Acceptance of wealth inequality	**0.16**	**0.45**	**0.13**	(0.71)							
5.	Collective action intention	**-0.08**	**-0.22**	0.02	**-0.28**	(0.80)						
6.	Age	-0.02	-0.05	**-0.27**	**0.08**	**-0.12**	–					
7.	Gender	**0.10**	-0.06	-0.03	**-0.07**	**0.13**	0.04	–				
8.	Marital status	**0.16**	0.05	**-0.10**	**0.10**	**-0.13**	**0.46**	0.04	–			
9.	Education level	**0.50**	**0.25**	**0.23**	**0.10**	0.03	**-0.39**	0.02	**-0.14**	–		
10.	Identification with Hong Kong	-0.01	**0.09**	**0.30**	0.03	**0.11**	**0.11**	0.07	0.07	-0.01	(0.71)	
11.	Past collective action	-0.01	**-0.16**	0.02	**-0.20**	**0.31**	**-0.14**	0.05	-0**.08**	**0.14**	0.07	–
*M*		3.49	2.89	3.44	2.59	2.61	2.95	0.52	0.66	2.44	3.83	0.14
*SD*		1.62	1.02	0.86	0.99	1.10	1.17	0.50	0.47	0.62	0.88	0.35

*N = 866. Significant correlations (*p* < 0.05) are in bold. Reliabilities (Cronbach’s alpha) are in parentheses. Personal income, age, and education level were ordinal variables. Gender, marital status, and past collective action were dummy variables (see section “Materials and Methods”).*

Multivariate findings revealed an absence of income main effect (*b* = 0.00, *p* = 0.888), but a presence of mobility main effect on AWI (*b* = 0.39, *p* < 0.001; Table 2). Results also showed that income was not related to CAI (*b* = -0.03, *p* = 0.212), but there was a significant and negative relation between AWI and CAI (*b* = -0.23, *p* < 0.001). The general indirect effect of income on CAI via AWI was not significant [indirect effect = -0.001, 95% CI (-0.012, 0.001)]. H1 was not supported.

**Table 2:** Conditional indirect effect of income on CAI through AWI.

Predicting AWI					
		***b***	***SE***	***p***	
**Predictor**					
Income		0.00	0.02	0.888	
Mobility		0.39	0.03	<0.001	
FTP		-0.01	0.04	0.754	
Income × mobility		0.00	0.02	0.831	
FTP × income		0.04	0.02	0.079	
FTP × mobility		0.01	0.03	0.857	
FTP × income × mobility		0.05	0.02	0.010	
**Covariate**					
Age		0.07	0.03	0.020	
Gender		-0.07	0.06	0.239	
Marital status		0.05	0.07	0.449	
Education level		0.07	0.06	0.257	
Identification with Hong Kong		0.00	0.04	0.940	
Past collective action		-0.35	0.09	<0.001	

**Predicting CAI**					

**Predictor**					
Income		-0.03	0.03	0.212	
AWI		-0.23	0.04	<0.001	
**Covariate**					
Age		-0.04	0.04	0.210	
Gender		0.23	0.07	0.001	
Marital status		-0.15	0.08	0.075	
Education level		0.02	0.07	0.773	
Identification with Hong Kong		0.12	0.04	0.002	
Past collective action		0.77	0.10	<0.001	

**Value of mobility**	**Value of FTP**	**Conditional indirect effect**	***SE***	**95% CI_lower_**	**95% CI_upper_**

**Bootstrapping results of conditional indirect effect**					
-1 *SD*	-1 *SD*	-0.004	0.011	-0.025	0.018
-1 *SD*	*M*	-0.002	0.007	-0.015	0.013
-1 *SD*	+1 *SD*	0.001	0.009	-0.020	0.018
*M*	-1 *SD*	0.007	0.008	-0.008	0.025
*M*	*M*	-0.001	0.006	-0.012	0.011
*M*	+1 *SD*	-0.009	0.007	-0.024	0.004
+1 *SD*	-1 *SD*	0.018	0.013	-0.005	0.047
+1 *SD*	*M*	0.000	0.008	-0.016	0.016
+1 *SD*	+1 *SD*	-0.018	0.009	-0.038	-0.002

*Ms and SDs of Mobility (perceived upward mobility) and FTP (future time perspective) are reported in Table 1. Income, personal income; AWI, acceptance of wealth inequality; CAI, collective action intention.*

**FIGURE 2:**
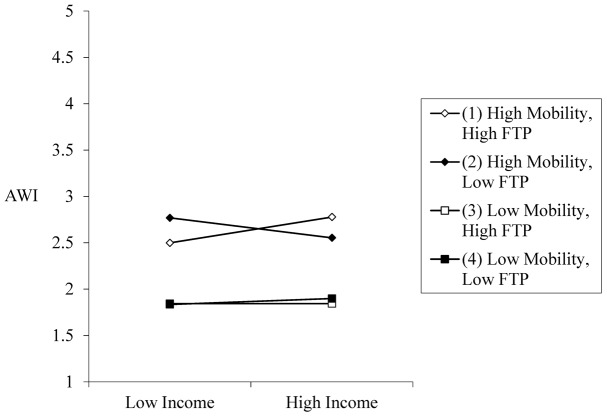
Relation between Income (personal income) and AWI (acceptance of wealth inequality) as a function of Mobility (perceived upward mobility) and FTP (future time perspective).

The two-way interaction effect between income and mobility (*b* = 0.00, *p* = 0.831) on AWI was not significant. H2 was also not supported. Nevertheless, there existed a three-way interaction effect among income, mobility, and FTP on AWI (*b* = 0.05, *p* = 0.010). When FTP was relatively high (one *SD* above the mean), there was a two-way interaction between income and mobility on AWI (*b* = 0.05, *p* = 0.045). When FTP was low, this two-way interaction did not emerge (*b* = -0.06, *p* = 0.066). Specifically, simple slope analysis showed that income was positively related to AWI (*b* = 0.08, *p* = 0.019) when mobility and FTP were relatively high (one *SD* above the means; Figure 2)^3^. In contrast, the income-AWI relation was not observed when mobility and FTP were low (*b* = 0.02, *p* = 0.637), when mobility was high and FTP was low (*b* = -0.08, *p* = 0.099), and when mobility was low and FTP was high (*b* = 0.00, *p* = 0.958). H3 was supported.

Given the presence of this three-way interaction effect, a significant conditional indirect effect of income on CAI through AWI emerged: only when mobility and FTP were relatively high, higher income was associated with higher AWI and in turn, lower CAI [indirect effect = -0.018, 95% CI (-0.038, -0.002)]. These findings corroborated H4.

## Discussion

In this study of acceptance of wealth inequality (AWI), we have advanced a model that describes personal income, perceived upward mobility (mobility), and future time perspective (FTP) as antecedents of AWI, and collective action intention (CAI) as an outcome. Our findings reveal a three-way interaction effect among income, mobility and FTP on AWI, and related, a conditional indirect effect of income on CAI via AWI. These data contribute to the literature on AWI as well as that on CAI.

### Prediction of Acceptance of Wealth Inequality

Wealth inequality reflects profound status difference in a society. Inferred from the social identity literature (Tajfel and Turner, 1979; Hogg, 2005), one might argue that rich people have higher AWI than poor people. While income and AWI are indeed positively correlated at zero-order level, multivariate findings indicate that they are actually related in a more subtle way; the income effect on AWI needs to be considered in conjunction with mobility and FTP.

According to socioemotional selectivity theory (Carstensen, 1991, 2006; Carstensen et al., 1999), compared with the counterparts with limited FTP, people with open-ended FTP have a stronger instrumental focus. From this perspective, the latter group should be more influenced by instrumental matters including personal income and perceived upward mobility. In addition, lower mobility is related to lower AWI (Shariff et al., 2016). Thus, the positive association of income with AWI is offset by lower mobility. Altogether, the positive income-AWI relation is only observed among individuals who perceive they can move up the social hierarchy, and that they have much future time left.

The importance of mobility in AWI points out the alarming consequence of being stuck in the social hierarchy. Efforts aimed to modulate mobility may build on the literature on perceived employability. Research has revealed adaptability (e.g., boundaryless mindset), career identity (e.g., career self-efficacy), human (e.g., education level), and social capital (e.g., networking) as contributing factors of employability (Fugate et al., 2004; McArdle et al., 2007), which may promote mobility (Jetten et al., 2008). These suggest that efforts may elevate mobility through addressing individuals’ sense of adaptability, career identity, human, and social capital.

Future time perspective is also influential to AWI. Our findings indicate a stronger association between income and AWI when people with open-ended FTP have higher mobility, and this could be attributed to the positive linkage between FTP and instrumental motives (Carstensen, 1991, 2006; Carstensen et al., 1999). Two streams of follow-up research are called. First, to better demonstrate causation, future studies may manipulate individual’s FTP (extended vs. limited; see Fung et al., 1999; Fung and Carstensen, 2004), and address whether those with extended FTP may show high levels of AWI, depending on their income and mobility. Secondly, our premise about the significance of FTP in the impacts of income and mobility on AWI concerns instrumental motives, which opens up the possibility that other parameters with implications for instrumental motives may also qualify the impacts of income and mobility. Specifically, converging evidences showing that the moderating role of system justification ideologies (Willis et al., 2015), money attitudes (Lim et al., 2003), and materialism orientation (Richins and Dawson, 1992) mirror that of FTP can substantiate the claims made here.

### Prediction of Collective Action Intention

Considering CAI as an outcome of AWI, we have revealed that the income–AWI–CAI linkage is conditional upon perceived upward mobility and FTP, as these two parameters moderate the relation between income and AWI. To further our understanding of the income–AWI–CAI linkage, it is useful to address what may moderate the association between AWI and CAI. Supplementary findings revealed that the AWI–CAI association was not qualified by FTP (*b* = -0.01, *p* = 0.697). However, there may be a three-way interaction effect among AWI, FTP, and social support for collective action on CAI, as extrapolated from the socioemotional selectivity theory (Carstensen, 1991, 2006; Carstensen et al., 1999). People with limited FTP are highly concerned about social connections, and tend to avoid conflicts with significant others. Even if having low AWI, they may actually show low CAI if they perceive that their significant others are not supportive of their participation in collective action. Hence, among them, the negative association between AWI and CAI should be attenuated when support is low than when support is high. Relatively speaking, the association between AWI and CAI should be less dependent on perceived support among people with open-ended FTP.

The contemporary collective action literature focuses on the dynamic between advantaged and disadvantaged groups, and studies how group-related variables influence CAI (van Zomeren et al., 2011; van Zomeren, 2013). In establishing the interplay among personal income, perceived upward mobility, and FTP on CAI (through AWI), the current research represents a revisit of the individual perspective on collective action, which has been less stressed in recent years. In the United States, President Donald Trump has recently advocated a tax reform. This reform is highly controversial as it may spur wealth inequality (Johnson, 2017). Researchers may take a group perspective and address how group-related variables such as group-based anger predict collective action for/against this reform. Alternatively, based on our findings, researchers may adopt an individual perspective and study collective action as an outcome of acceptance of tax reform, which may depend on income, mobility, and FTP.

### Limitations and Future Directions

This study has several issues that need to be acknowledged. First, we tested mediation using cross-sectional data. Secondly, we only had data on CAI, but did not assess future collective action as the ultimate criterion. Nevertheless, the attitude-behavior literature has documented that behavioral intention is a significant predictor of future behavior (Ajzen, 1991). Hence, our findings of CAI are informative to the understanding of future collective action against wealth inequality.

Another issue of this work is that the scales used in the telephone survey only had 2–three items. The scales showed acceptable internal consistency (α > 0.70). But we note that replications using scales with more items will be useful.

From our understanding, income or generally financial matter is a very sensitive topic in Hong Kong. To facilitate responding and avoid missing data in our telephone survey, we asked income in terms of ranges, a common practice in local studies (Chou, 2010; Li et al., 2011). Our income ranges were based on Li et al. (2011) and Hong Kong Population Census (Hong Kong Census and Statistics Department, 2012). It is also worth noting that some relevant studies in the United States also used income ranges (e.g., Wakslak et al., 2007; Chow and Galak, 2012). Notwithstanding, future research should consider asking the exact amount of income.

Here, we use the term acceptance of wealth inequality. For consistency, our response options were “totally not accept” and “totally accept.” A caveat must be highlighted: when being asked whether they “accepted” the factual information about wealth inequality in Hong Kong, the participants might be thinking about the accuracy of the information, rather than the fairness of the situation, as intended. At the same time, we need to point out that there is no consensus on terminology and operationalization in the literature. To illustrate, Savani and Rattan (2012) studied acceptance of wealth inequality, but they operationalized this construct in terms of disturbance by wealth inequality (1 = not at all disturbed, 7 = extremely distributed). Shariff et al. (2016) studied tolerance for income inequality. The sample item read “I think that the current amount of income inequality in the United States is unacceptable” (1 = strongly disagree, 7 = strongly agree). Future meta-analytic reviews should look into the implication of terminology and operationalization in the concerned effect size.

The current survey did not capture system justification ideologies (Jost and Hunyady, 2005), which should be included to better reveal the unique importance of income, mobility, and FTP in AWI (and CAI). Also, system justification research has showed that anticipated future of the society may influence inequality legitimization (Laurin et al., 2013). At the same time, we have revealed the impact of FTP, or perceived time left in life (Carstensen, 1991, 2006; Carstensen et al., 1999) on AWI. Altogether, the role of “future consideration” in AWI seems an interesting topic and merits more exploration.

Chinese culture values emotional control and moderation in responding (Soto et al., 2005). This could be why our Hong Kong sample did not report particularly low acceptance of inequality when they were poor, perceived low mobility, and had high FTP. It is possible that an alternative pattern of the three-way interaction will be observed when researchers adopt a sample from another culture. In any event, based on our extrapolation of the literature, we have come up with the present set of predictions. Our data, which involved a large community sample, has lent support to our arguments. Also noteworthy is that past collective action was included as a controlling variable. Past behavior is a strong predictor of attitude and behavioral intention (AWI and CAI in our case; Festinger, 1957; Ajzen, 1991). Our findings about the significance of income, mobility, and FTP thus seem robust and striking.

## Conclusion

Wealth inequality has grown in prominence on social agendas across the world. The present study addresses this timely issue. We have provided the first set of findings that illustrate a three-way interaction effect among personal income, mobility and FTP on AWI, and related, a conditional indirect effect of income on CAI via AWI. The confirmation of these associations has pointed to the relevance of an individual-level instrumental perspective to the understanding of AWI and CAI.

## Ethics Statement

This study was carried out in accordance with the recommendations of the Survey and Behavioural Research Ethics Committee at the Chinese University of Hong Kong. Verbal informed consents were obtained from all participants in the telephone survey. The study and protocol were reviewed and approved by the Faculty Sub-committee under the Survey and Behavioural Research Ethics Committee at the Chinese University of Hong Kong.

## Author Contributions

GC designed the study, analyzed the data, and wrote the manuscript. DC designed and conducted the study, and wrote the manuscript. DY designed the study and wrote the manuscript.

## Conflict of Interest Statement

The authors declare that the research was conducted in the absence of any commercial or financial relationships that could be construed as a potential conflict of interest.

## Footnotes

1 From an economic perspective, income and wealth are related but different, and economic inequality is a broader concept that includes both wealth inequality and income inequality. In the psychology literature on the concerned topic, however, “wealth inequality” is used interchangeably with “income inequality” and “economic inequality” (Norton and Ariely, 2011; Tyler, 2011). Following Savani and Rattan (2012), we choose to use “wealth inequality.”

2 A recent government report showed the following monthly income distribution (Hong Kong Census and Statistics Department, 2017a): below $10000 (18.5%), at least $10000 and below $20000 (42.9%), at least $20000 and below $30000 (16.5%), at least $30000 and below $40000 (8.1%), and $40000 or above (14%). Our data mirrored the official counterpart and reflected a full range of variability in wealth in Hong Kong.

3 Three-way interaction effect is complex and there are several ways of looking at it. We report and illustrate our results following our hypotheses. We can provide alternative plots to illustrate the interaction effect upon request.
